# Cognitive behavioural group therapy versus mindfulness-based stress reduction group therapy for intimate partner violence: a randomized controlled trial

**DOI:** 10.1186/s12888-020-02582-4

**Published:** 2020-04-19

**Authors:** Merete Berg Nesset, Mariela Loreto Lara-Cabrera, Johan Håkon Bjørngaard, Richard Whittington, Tom Palmstierna

**Affiliations:** 1 Forensic Department and Research Centre Brøset, St. Olav’s Hospital, Trondheim University Hospital, Trondheim, Norway; 2 Faculty of medicine and health sciences, Department of Mental Health, Norwegian University of Science and Technology (NTNU), Trondheim, Norway; 3 Tiller Community Mental Health Centre, St. Olav’s Hospital, Trondheim University Hospital, Trondheim, Norway; 4 Department of Research and Development, St Olav’s Hospital, Trondheim University Hospital, Trondheim, Norway; 5 Department of Public Health and Nursing, Norwegian University of Science and Technology (NTNU), Trondheim, Norway; 6 Department of Clinical Neuroscience, Centre for Psychiatric Research, Karolinska Institutet, Stockholm, Sweden

**Keywords:** Batterers, Cognitive-behavioural group therapy, Domestic violence, Intervention, Intimate partner violence, Mindfulness-based stress reduction, Randomized controlled trial, Treatment effectiveness

## Abstract

**Background:**

Violence in close relationships is a global public health problem and there is a need to implement therapeutic programs designed to help individuals who voluntarily seek help to reduce recurrent intimate partner violence. The effectiveness of such interventions in this population remains inconclusive. The aim of the present study was to compare the effectiveness of cognitive-behavioural group therapy (CBGT) vs mindfulness-based stress reduction (MBSR) group therapy in reducing violent behavior amongst individuals who are violent in intimate partnerships and who voluntarily seek help.

**Methods:**

One hundred forty four participants were randomized using an internet-based computer system. Nineteen withdrew after randomization and 125 participants were randomly assigned to the intervention condition (CBGT, *n* = 67) or the comparator condition (MBSR, *n* = 58). The intervention condition involved two individual sessions followed by 15 cognitive-behavioural group therapy sessions. The comparator condition included one individual session before and after 8 mindfulness-based group sessions. Participants (*N* = 125) and their relationship partners (*n* = 56) completed assessments at baseline, and at three, six, nine and twelve months’ follow-up. The pre-defined primary outcome was reported physical, psychological or sexual violence and physical injury as measured by the revised Conflict Tactics Scale (CTS2).

**Results:**

The intent-to-treat analyses were based on 125 male participants (intervention group *n* = 67; comparator group *n* = 58). Fifty-six female partners provided collateral information. Baseline risk estimate in the CBGT-group was .85 (.74–.92), and .88 (.76–.94) in the MBSR-group for physical violence. At 12-months’ follow-up a substantial reduction was found in both groups (CBGT: .08 (.03–.18); MBSR: .19 (.11–.32)).

**Conclusion:**

Results provide support for the efficacy of both the cognitive-behavioural group therapy and the mindfulness-based stress reduction group therapy in reducing intimate partner violent behavior in men voluntarily seeking treatment.

**Trial registration:**

NCT01653860, registered July 2012.

## Background

Violence in close relationships is a global public health problem and there is a need to implement therapeutic programs designed to help perpetrators, including those who voluntarily seek support, to end their engagement in recurrent intimate partner violence (IPV).

Recent studies have reported that in intimate partner homicide cases, earlier incidents of intimate partner violence had been registered by the police and/or in the health- and social care services [1, 2]. This calls for a coordinated approach to prevent recurrent family violence before it escalates where both the police and health and social services are essential [3]. An important part of the health services’ role in protecting the victims, is to offer therapy and support to the perpetrator of intimate partner violence [4].

Cognitive behavioural therapy is commonly used to address dysfunctional anger and violent behaviour among intimate partners, working with dysfunctional beliefs and appraisals that operate to generate negative affect, motivation, behaviour, and physiological responses. Cognitive behavioural group therapy (CBGT) can help individuals to recognise distorted patterns of thinking and emotional regulation problems by enabling observation of other group participants which can help them to understand the function of their violence as a way to resolve stressful situations and emotional distress [5–7].

A recent systematic review found that randomized controlled trials investigating CBGT have focused mainly on mandatory group interventions delivered within the prison service and in outpatient settings, as well as combining group participants who are involuntarily assigned to treatment with group participants seeking treatment on their own initiative [8]. Even though some studies have found positive treatment effects of CBGT for partner violent behaviour [9–11], very few randomised controlled trials have specifically investigated patients voluntarily seeking treatment as a distinct group [8]. Treatment motivation and engagement and the ability to change may be different between those court ordered to therapy versus those voluntarily undergoing therapy [12]. So far, only two randomized controlled trials have been conducted which suggested that CBGT is effective to support voluntary and self-referred participants in reducing violence [10, 11]. Palmstierna et al. [11] found reductions in self-reported violence in the CBGT group (*n* = 26) as compared to the waiting list group (*n* = 11) 15 weeks after baseline. Taft et al. [10] found greater reductions in reported physical and psychologically intimate partner violence in the CBGT group (*n* = 67) at 6 months’ follow-up after baseline as compared to treatment as usual (TAU, *n* = 68). Although these results are promising, the research base is still small and more randomized controlled trials are needed to evaluate the clinical efficacy of CBGT in this group.

The present randomized controlled trial evaluated the effects of CBGT on men’s violent behaviour towards their female partners in a voluntary sample in Norway. The study adopted mindfulness-based stress reduction (MBSR) as an active control condition because it was the only feasible comparator intervention in the region. MBSR uses methods derived from meditation and yoga to counteract stress and create a balance of body and mind and its effectiveness has been reported in earlier studies [13, 14]. Both interventions were given in a group format. The primary aim was to investigate if participants receiving CBGT would be less likely to report change in violent behaviour at 1 year follow-up compared to an active comparator group. We hypothesised that the participants in the CBGT-group would have greater reductions in violent behaviour compared to an active comparator group. The secondary aim was to investigate if the participants in the CBGT-group would improve reported mental health outcomes and emotion regulation at one-year follow-up as compared to an active comparator group. The secondary outcomes in the study will be reported in a separate paper (Nesset et al., submitted).

## Methods

### Study design and population

**Fig. 1:**
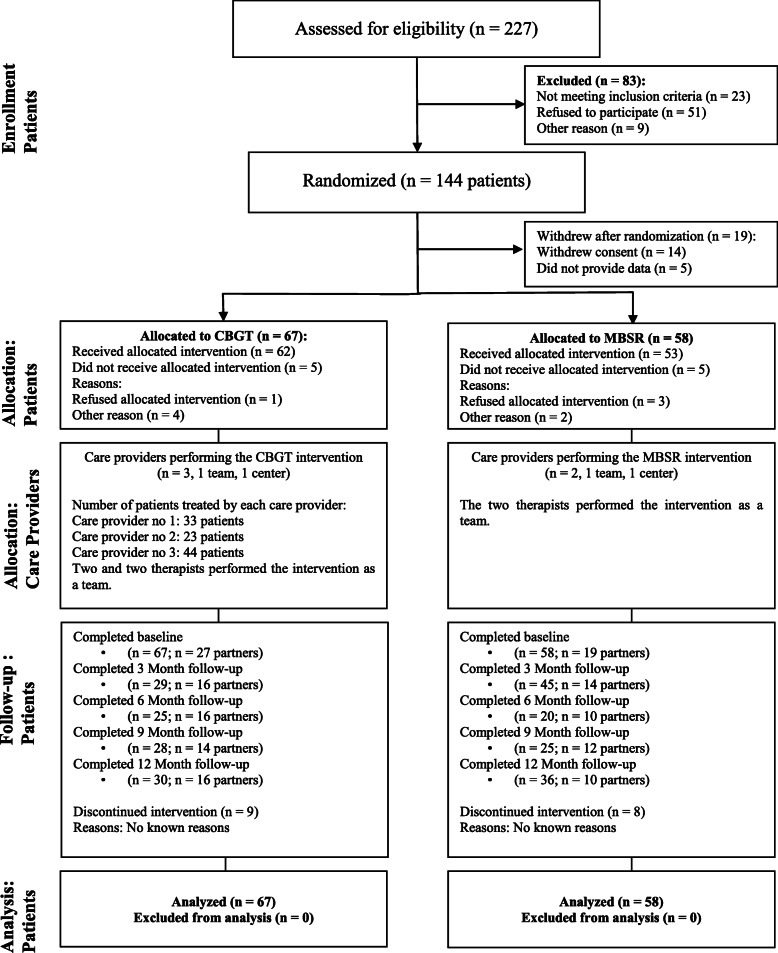
Flow diagram of participant enrolment, allocation, follow-up and analysis

The randomized controlled trial was conducted between July 2012 and May 2018. The patients were randomized to either a manualized CBGT condition or a comparator condition, MBSR. An active treatment comparison was chosen as the control condition [15]. We judged it unethical to withhold treatment from the control group because the sample consisted of individuals presenting problems with anger and violent behaviour towards others. Also, previous research has highlighted the need for, and importance of future studies comparing active treatments for intimate partner violence [10, 16]. All consecutive voluntary referrals by general practitioners of partner-violent adult men (*N* = 227) to St. Olav’s University Hospital, Forensic Department and Research Centre Brøset’s therapy program for aggressive and violent behavior, were assessed for eligibility. The inclusion criteria for participation in the study were that participants were male, aged 18 years or older, were violent towards a current partner or ex-partner, understood and spoke Norwegian fluently, admitted to having problems with anger and violence towards their female partner and provided written informed consent for study participation. Patients were excluded from joining the study if they were violent towards non-partners only, had current uncontrolled psychotic symptoms, or were using drugs or alcohol to a degree that it was impossible to be sober during treatment sessions. Those who met the necessary criteria and agreed to participate (*n* = 144) were randomly assigned to one of the two treatment conditions. Figure 1 describes the participant flow from recruitment to study completion. Partners of participating perpetrators were also eligible if they agreed to take part (*N* = 56).

### Recruitment and procedures

All participants (perpetrators and partners) received written and verbal information about the study prior to recruitment. Postgraduate hospital staff conducted the consent procedures, and written consent was obtained from the participants prior to approaching partners and beginning baseline assessments. Provided the patients gave their written consent, their partners were contacted by phone to inform them about the study and to ask for their consent to participate in it. The partners were assured that their responses to the questionnaires would remain confidential. Those who agreed sent a written consent by mail to the hospital. In cases where any participant subsequently wished to remove their consent to participate, they approved inclusion of already collected anonymous data in the study.

### The intervention and the comparator groups

Two active interventions were compared in this study: Cognitive behavioural group therapy (intervention group) versus mindfulness-based stress reduction group therapy (comparator group). Both interventions were delivered in an outpatient health service setting.

#### Cognitive behavioural group therapy (CBGT)

Participants in the intervention group received two individual sessions followed by 15 cognitive-behavioural group therapy sessions (total 30 h). The groups consisted of four to six patients in each group The key principles used in the CBGT focused on establishing a therapeutic relationship, behavioural change strategies, cognitive restructuring, modification of core beliefs and schemas, and the prevention of relapse and recurrent violence [6, 17]. The first five sessions were psychoeducational and focused on dysfunctional anger and how information processing was tied to affective, motivational and behavioural responses in humans. Information about the consequences of domestic violence on the victims (partner and children) in the family was discussed and the patients’ pattern of violence was explored by identifying typical risk situations. The remaining ten sessions all began with a review of a practice assignment (e.g. practice in communication and partner conflict resolution skills). Each patient presented a violent episode to the group, which was analysed by exploring negative automatic thoughts and maladaptive beliefs activated in the particular situation, and by reviewing the evidence for and against these thoughts and beliefs and considering alternative interpretations of the situation that led to violence. In addition, by practising on taking time-outs in violence risk situations, the patients were trained to accept and cope with negative emotions without acting them out. Toward the end of the group therapy, the patients created action plans for future conflict risk situations.

#### Mindfulness-based stress reduction group therapy (MBSR)

The comparator condition consisted of one individual session before and one session after eight group sessions of mindfulness-based stress reduction group therapy (16 h) [18, 19]. The aim of the comparator condition was to develop the skills of noticing the presence of negative thoughts without avoiding them. Also, it aimed to enhance consciousness of body sensations and mood in anger provoking situations, awareness of the interaction with others in high-arousal situations and learning of skills to manage negative emotions without acting them out violently. The key principles used in the MBSR focused on techniques derived from meditation and yoga to counteract stress and create a balance of body and mind. The groups size varied between four to six patients. The patients were expected to practice new skills every day at home between the sessions [18, 19].

#### Group therapists

Three psychiatric nurses delivered the CBGT and specialists in clinical psychology and education delivered the MBSR. Both interventions were manualized, and all five therapists had formal education and training in cognitive behaviour therapy and mindfulness respectively. Adherence to the study protocol was monitored through regular meetings between the therapists and the researchers. Furthermore, the therapists detailed the content of each group session in clinical records. A research assistant monitored the clinical documentation.

### Outcome measurements

The baseline assessments were completed by the participants based on self-report, guided by the hospital staff after the randomization, but completed without assistance at the follow-up assessments. The primary outcome was assessed at baseline and at 3, 6, 9 and 12 months’ after baseline. For the follow-up assessments, the participants (perpetrators and partners) could choose to self-report electronically or by paper. The participants who did not return their questionnaires within a week were contacted by SMS, encouraging them to convey their answers. If they still did not answer, the follow-up questionnaires were posted again up to two times. The respondents were also encouraged to contact the study research assistant if they needed help to complete the questionnaires.

### Primary outcome

The pre-defined primary outcome was change in violent behavior at 12 months’ follow-up. Violence was assessed over the preceding 3 months, as reported by the male participants and their female partners at baseline and at 3, 6, 9 and 12 months’ follow-up, using the Norwegian version of the revised Conflict Tactics Scales (CTS2) [20, 21]. Data was collected from the CTS2 subscales physical violence (12 items), physical injury (6 items), psychological violence (10 items), and sexual violence (3 items). The CTS2 is a widely used instrument and measures four dimensions of intimate partner violence, i.e. the extent of physical, psychological and sexual violence and resulting physical injury [22]. Cronbach’s alpha for the current sample (both conditions combined) was .89. The response categories for each item range from 0 to 7 and measure incidence over the previous 3 months (0 = never happened, 1 = happened once, 2 = happened twice, 3 = happened 3–5 times, 4 = happened 6–10 times, 5 = happened 11–20 times, 6 = happened more than 20 times). The standard CTS2 has a seventh score (7 = never happened in the last 3 months, but has happened before). In this study the seventh score was not used since it was focused upon behavior over the preceding 3 months. The CTS2 violent behaviour outcome was dichotomized, where 0 was defined as no reported violence and 1 was defined as one or more episodes of any type of violence. The Norwegian version of the CTS2 has been used in a Norwegian student population [21].

### Sample size

The power calculation was based on an assumption of a Poisson distributed number of violent behaviour events per individual on the CTS2. We expected some within-individual clustering but we did not find any estimates of such clustering or the actual level of violence events in previous studies. Hence, we assumed a within-individual clustering of 30%. With a statistical significance level of 5% when comparing the two groups, we anticipated being able to detect a 20% difference (10 events vs 8 events) with 80% power with a sample size of 134 (67 individuals in each study arm).

### Randomization

The participants were allocated to intervention using an Internet-based computer program provided independently by the Research Trial Service Centre at the Norwegian University of Science and Technology (www.webcrf.medisin.ntnu.no). A block randomization procedure (blocks of 10) with no stratification was used, and the participants were informed of their group allocation immediately after the randomization. Those involved in the trial were blinded to the block sizes.

### Blinding

Participant and partner data had unique codes and the analyst was blinded to the identity of participants until finalizing the results. The researchers were blinded to the randomization procedure.

### Statistical analyses

**Fig. 2:**
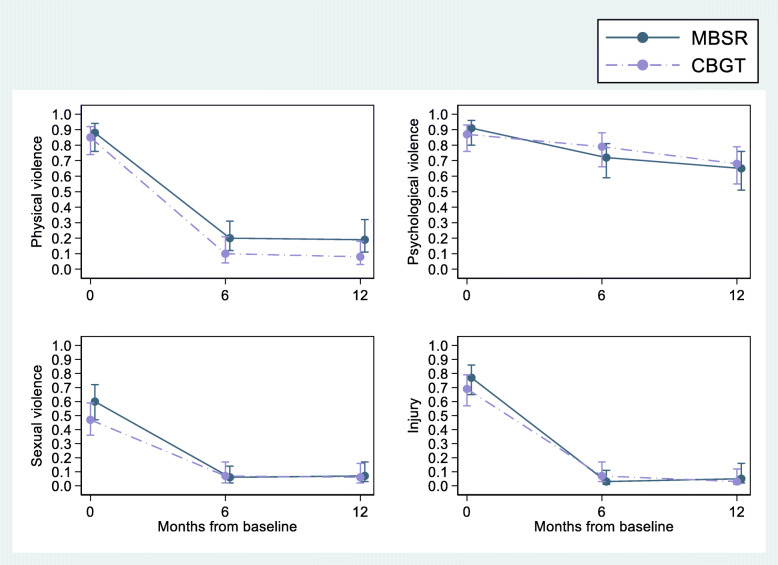
Estimated proportion of any incidents of violence last 3 months as measured by the CTS2. With 95% Confidence intervals (vertical lines) as a function of months from baseline. Estimates based on GEE logistic models

The descriptive analyses of baseline characteristics were performed with IBM Corp. SPSS, version 23.0 (IBM Corp. Released 2015. IBM SPSS Statistics for Windows, Version 23.0. Armonk, NY: IBM Corp.). The estimation of the effect of treatment on changes in intimate partner violent behaviour over time was conducted according to the intention-to-treat principle. The primary outcome was analyzed with STATA (StataCorp. 2017. Stata Statistical Software: Release 15. College Station, TX: StataCorp LLC). Due to a skewed distribution of violent events, we dichotomized the outcome into either ‘any violence’ or ‘no violence’. We combined the time points 3 and 6 months’ follow-up, and 9 and 12 months’ follow-up, because of lower response rates during some of the intermediate assessments. Each follow-up wave was added to the model as a dummy variable (3–6 months and 9–12 months and with baseline as reference). In order to investigate differences between the groups during follow-up, we included interaction terms between group allocation and each registration time point. We estimated the proportion of any intimate partner violence according to time and intervention with 95% confidence intervals at each assessment, using a generalized estimating equation (GEE) model with a logit function (Fig. 2). To alleviate underreporting we compared the participant-scores and the partner-scores on each of the CTS2-items in our calculations and used the larger of the two individual item responses, i.e. the highest reported incidence of violence was included in the analyses.

#### Sensitivity analyses

We investigated possible differences in loss to follow-up between the groups with a linear regression model where the outcome variable was number of responses during follow-up. The concordance between clients and partners was assessed with a logistic regression analysis. Based on the distribution of the measure of psychological violence, we estimated the mean value of psychological violence between the two interventions based on a linear mixed model. We have also presented the results of an analysis of the outcome measures without any dichotomization, based on a linear mixed model. The linear and logistic mixed models use all the information available and is less sensitive for outcome based missing during follow-up compared to traditional methods [23, 24]. We also analyzed the data in a similar way using client only responses (see Additional file 1).

## Results

### Recruitment and study attrition

Participant flow from recruitment to study completion according to the CONSORT guidelines is presented in Fig. 1 [25]. Of the 227 men who were assessed for eligibility, 144 entered the study and were randomized. Nineteen participants withdrew after randomization: Fourteen participants dropped out before start of treatment and five did not return any questionnaire and hence, could not be included in the study. A total of 125 male participants returned the CTS2 questionnaire and were included in the intent-to-treat analyses, with 67 in the CBGT group and 58 in the MBSR group. Fifty-six female partners agreed and provided collateral information.

### Baseline characteristics

The baseline characteristics for the intent-to-treat sample is displayed in Table 1. More of the participants in the CBGT-group lived with their own children (61%) than those in the MBSR-group (44%). Also, more participants in CBGT (81%) than in MBSR (69%) had full time work. CTS2 at baseline indicates that physical and psychological violence was the most frequently reported type of violence, with 57 (85%) in the CBGT-group and 50 (86%) in the MBSR-group reporting physical violence, and 58 (87%) in the CBGT-group and 52 (90%) in the MBSR-group reporting psychological violence. At baseline, 46 (69%) in the CBGT-group and 44 (76%) in the MBSR-group reported physical injury resulting from violence. Moreover, 31 (47%) in the CBGT-group and 34 (59%) in the MBSR-group reported sexual violence at baseline.

**Table 1:** Baseline characteristics for the Intent-to-Treat sample by treatment condition. Values are presented as mean and standard deviation (SD) or proportion (%)

Variables	CBGT		MBSR	
	N^a^	%	N^a^	%
**Age**				
Age, n, mean	63	38.02	58	35.66
Age (SD)		(8.6)		(10.3)
**Higher education**	17	27%	12	22%
**Mother tongue**	63		58	
Norwegian, n (%)	57	91%	53	91%
**Family situation**	62		57	
Living with intimate partner, n (%)	33	67%	29	67%
Do not have children, n (%)	12	19%	14	25%
Living with own children, n (%)	38	61%	25	44%
Have children, but do not live with them, n (%)	10	16%	14	25%
Living with partners children, n (%)	10	16%	5	9%
**Employment**	62		55	
Unemployed, n (%)	8	13%	8	15%
Full time work, n (%)	50	81%	38	69%
Part time work, n (%)	4	7%	9	16%
**CTS2 at baseline**^b^	67		58	
Any physical violence	57	85%	50	86%
Any psychological violence	58	87%	52	90%
Any sexual violence	31	47%	34	59%
Any physical injury	46	69%	44	76%

*SD* Standard deviation, *CBGT* Cognitive Behaviour Group Therapy, *MBSR* Mindfulness-Based Stress Reduction, *CTS2* Conflict Tactics Scales Revised

^a^N, number varies due to missing values

^b^ As reported by perpetrator and partner

Table 1 Baseline characteristics for the Intent-to-Treat sample by treatment condition. Values are presented as mean and standard deviation (SD) or proportion (%).

### Treatment attendance

For CBGT, 62 (92.5%) participants received the allocated intervention (see Fig. 1). For MBSR, 53 (91.4%) participants received the allocated intervention. The number of participants who discontinued the intervention were 17; 9 (14.5%) for CBGT and 8 (15.1%) for MBSR respectively. Of those who dropped out of the CBGT- group, six did so between the individual sessions and group attendance. In the MBSR- group five participants dropped out before group attendance. The mean difference in number of valid responses did not deviate substantially between intervention and comparator groups (mean difference in number of responses between CBGT vs. MBSR was 0.13, with a 95% confidence interval [CI] -0.39 to 0.65).

### Follow-up and attrition

As seen in Fig. 1, 67 participants allocated to CBGT and 58 participants allocated to MBSR completed baseline assessments. At the study endpoint (12 months) 30 (44.7%) of the CBGT participants and 36 (62%) of the MBSR participants completed the assessment. Interim completion rates can be seen in Fig. 1. The mean response rate in the CBGT group was 2.6 responses and in the MBSR 3 responses.

### Primary outcome: violent behaviour

In terms of agreement between perpetrators and partners, the odds of the client reporting an incident when the partner reported one as well for each dimension were: psychological violence 3.86 times higher (95% CI 1.4 to 10.6); physical violence 11.2 times higher (95% CI 4.3 to 29.4); sexual violence 1.3 times higher (95% CI 0.3 to 4.9); physical injury to partner 11.7 times higher (95% CI 2.3 to 60.1). For sexual violence there was low concordance between client and partner reports, hence the results should be interpreted with caution.

Both the intervention and the comparator group showed substantial reductions in violent behaviour during 12 months follow-up, and no time-by-condition differences between the CBGT-group and the MBSR-group could be found. This finding was consistent across all dimensions of the primary outcome as measured by the CTS2, based on a combination of the highest reported level of violence from the participant or his partner over 1 year. Figure 2 displays the risk of any intimate partner violence according to time and intervention with 95% confidence intervals at each assessment, using a generalized estimating equation (GEE) model during the last 3 months. Additional file 1 presents mean differences (Supplementary Fig. 1, S-Table 1a–d) and odds ratios (S-Tables 2a – d) for any of the four different kinds of violence during the last 3 months as measured by the CTS2 at baseline (0), 6-month, and 12-month follow-up, clients and partners combined. In addition, for comparison we have provided odds ratios of client scores only (S-Tables 3 and 4a – d), and the combined client and partner mean scores at each time point for interested readers to see (S-Tables 5a – 8e).

For any physical violence the risk estimate at baseline was .85 (.74–.92) for the CBGT condition and .88 (.76–.94) for the MBSR condition. At 6-month follow-up the risk estimate was .10 (.04–.21) for the CBGT condition and .20 (.12–.31) for the MBSR condition. This effect held true at 12-month follow-up with a risk estimate of .08 (.03–.18) for the CBGT condition and .19 (.11–.32) for the MBSR condition.

The risk estimate at baseline with regard to any injury on partner was .69 (.57–.79) for the CBGT condition and .77 (.65–.86) for the MBSR condition. At 6-month follow-up the risk estimate was .07 (.03–.17) for the CBGT condition and .03 (.01–.11) for the MBSR condition. This effect held true at 12-month follow-up with a risk estimate of .03 (.01–.12) for the CBGT condition and .05 (.02–.16) for the MBSR condition.

For any psychological violence the risk estimate at baseline was .87 (.76–.93) for the CBGT condition and .91 (.80–.96) for the MBSR condition. At 6-month follow-up the risk estimate was .79 (.66–.88) for the CBGT condition and .72 (.59–.81) for the MBSR condition. At 12-month follow-up the risk estimate was .68 (.55–.79) for the CBGT condition and .65 (.51–.76) for the MBSR condition.

For any sexual violence the risk estimate at baseline was .47 (.36–.59) for the CBGT condition and .60 (.47–.72) for the MBSR condition. At 6-month follow-up the risk estimate was .07 (.02–.17) for the CBGT condition and .06 (.02–.14) for the MBSR condition. At 12-month follow-up the risk estimate was .06 (.02–.16) for the CBGT condition and .07 (.03–.17) for the MBSR condition. The results from the sensitivity analyses (see Additional file 1, S-Tables 1–4) were in line with and confirmed the results presented in Fig. 2. An analysis of client scores only was consistent with the scores from client and partner combined (see Additional file 1, S-Table 3 and 4a – 4d).

## Discussion

### Main findings

The primary aim was to investigate if participants receiving CBGT would be less likely to report violent behaviour at 1 year compared to those receiving MBSR. The findings failed to support the hypothesis that the participants in the CBGT-group would have greater reductions in violent behaviour compared to an active comparator group. The changes in the CBGT- group were also observed for the comparator group as well, so with similar reductions in both groups, it is not possible to conclude if both interventions are effective or both benefited from similar extraneous processes such as protective orders. The analysis was based on participants self- and partner-reported data and indicated that both in the CBGT and MBSR group physical and sexual violence was substantially reduced, and also physical injury on partners in both groups, with no difference between the two groups. One explanation for the similar reduction in reported violence in both groups might be a design issue in that our study has an active therapy as a comparator condition as reported by Wright et al. [26], which in our study was due to pragmatic reasons and is contrary to Palmstierna’s [11] approach for instance which used a waiting list comparator group. An active comparator control has the advantage of possibly being more credible to the participants. However, there are always particular challenges in demonstrating group differences when comparing two potentially effective therapies [15, 27]. It may be that the participants in our study could have experienced particularly stressful life circumstances at the time of admittance to the health service, and hence exert higher frequency and more serious violence than they normally would. Thus, regression to the mean may explain some of the reductions in violence during the 12 months follow-up.

The CBGT-group reported reduced violence during 12 months follow-up. These results are in line with the observations from three other randomized controlled trials of the effect of cognitive behaviour group therapy on intimate partner violence for voluntary patients [11], mandatory patients [9] and mixed groups [10]. This study and the other three have demonstrated a reduction in violence amongst men receiving CBGT, with varying changes in the comparator group. Current research indicates that MBSR is effective on a number of mental health conditions [13, 14, 26]. Therefore, the present study adopted MBSR as an active control condition. In contrast to CBT, MBSR does not focus on cognitive biases or the recognition and alteration of distorted patterns of thinking. MBSR rather develops the skills of noticing the presence of negative thoughts and feelings without avoiding them. However, both interventions shared awareness of a patient’s emotions, and this common focus might explain why we were unable to show a significant benefit of CBT when compared to MBSR. Similar to the MBSR-condition in our study, other therapies targeting emotion-regulation skills in combination with traditional CBT to manage aggressive feelings have been associated with reduced intimate partner violence [28].

With regard to psychological violence the reduction was less extensive in both groups compared to the other forms of violence. Psychological violence seems to be relatively difficult to address. Hence, a study period of 12 months like in this study may be too short follow-up time to achieve substantial changes. In comparison, a study of long term sustainability (4 to 7 years after therapy) of cognitive behaviour group therapy among men voluntarily seeking treatment, reported a substantial further reduction of psychological violence after therapy [6].

With respect to the generalizability of these findings, it should be highlighted that 22.4% (51 of the 227 who were eligible) refused to participate in the trial. Those refusing participation may have been more or less likely to benefit from either intervention. But it should be noted that all those who were eligible were voluntary self-referrals which suggests that all potential participants had an understanding of the detrimental consequences of their violence on the family and were motivated to seek therapy regardless of participation in the study. Also, with regard to the substantial reduction in violence in this study as compared to other comparable studies (see for instance [9, 29]), this difference could possibly be explained by a different selection of participants. In many studies [8, 29] a substantial part of the participants is legally mandated to therapy and thus probably not as motivated as participants voluntarily seeking help to reduce their violence. One could hypothesize that by selecting individuals admitting to having anger problems we introduced a biased sample who would respond positively to all kinds of therapy. However, over all, to date only a small number of studies have been able to prove a positive outcome of treatment, even for those voluntary seeking treatment (see for instance [4, 8, 11]). Further, a review of interventions to intimate partner violence perpetrators concluded that there is a lack of evidence on effective treatments for intimate partner violence in a European context [30]. Moreover, intimate partner violence is a worldwide public health problem, where those admitting to having anger problems constitutes a large part of this population. Hence, finding effective treatment to this group of perpetrators could possibly prevent a considerable number of repeated violence against partners and children [31].

With regard to future directions this study needs to be replicated using a larger sample size and among other populations of intimate partner violence perpetrators, like for instance women, individuals in same sex relationships, and those legally mandated to attend therapy. Convicted populations are likely to face bigger challenges in terms of changing their behaviour, but studies with this population are less likely to experience attrition to the same extent as those with voluntary participants as here. The active treatment chosen as the control condition in our study had the benefit of being credible to potential participants. However, the choice of comparison could possibly have made it more difficult to determine, as noted above, whether the reduction of violence found in our study was a result of the two interventions or simply a time effect [15]. Hence, the study needs to be replicated and tested with other types of therapies to determine if change in violent behaviour was a result of treatment. Future studies should include other methods for improving study retention in clinical trials to ensure a higher response rate (personal assistance when completing the questionnaires, reminders and other incentives for completing the assessments). Important in our study was that the MBSR had a shorter intervention period (8 weeks vs 15 weeks in CBGT) and still produced the same reduction in reported violence. From a public health perspective and with regard to balancing potential costs and benefits one could argue that MBSR would be a feasible alternative to the more extensive CBGT. Our study did not explore possible mechanisms of the observed behaviour change. Hence, investigating active components in both treatments would be valuable and an important next step in future research and development of treatment for IPV. Also, given the challenges in reducing psychological violence observed in our study, exploring common features in therapies like CBGT and MBSR as well as other therapies addressing emotion regulation and mind-body awareness may provide an important component in future developments of IPV interventions. Acceptance as a mechanism of behavioural change has recently been proposed to be an effective way to reduce violence. For instance, research on acceptance and commitment therapy has indicated that awareness and acceptance of emotion appears to reduce psychological and physical aggression [32]. Further, addressing substance use and traumatic experiences may be relevant in future developments of IPV interventions [10, 16].

### Strengths and limitations

A strength of the study was that the outcome assessment was based on both participant and partner reports where possible which enhances the reliability of this measurement. The allocation sequence was concealed from the persons randomizing the male participants to prevent selection bias. Furthermore, uptake of the intervention was good (90% in CBGT, 87% in MBSR). There were moderate levels of drop-out from treatment, but the rates were similar in both conditions and almost all the drop-out occurred prior to the group phase. Consequently, the drop-out is not likely to be connected with group allocation or outcome. The expertise of the therapists in both groups was similar in terms of training in CBGT and MBSR respectively. Even though neither participants nor therapists could be blinded to treatment allocation, both groups received an active intervention. Therefore, expectations of improvements would have been present in both groups. Also, all the assessors involved in data analysis were blinded to group assignment.

A limitation in this study was that for sexual violence the outcome data from partners were incomplete, hence the results were mainly based on the male participants’ scores. Furthermore, the participant and partner scores did not correspond on this dimension, and the results should therefore be interpreted with caution. Even though the participants were given incentives and reminders for responding to the questionnaires, there was a substantial attrition during follow-up, especially in the CBGT group at 3-month follow-up, which possibly introduced bias due to incomplete outcome data. However, the missing response did not differ substantially between the treatment groups. Also, in a sensitivity analysis, we used a linear mixed model which is less susceptible to outcome based missing [23], and these results were in line with the results from the main analysis.

## Conclusion

The present study suggests a strong reduction in intimate partner violent behaviour over 1 year in men allocated to CBGT or MBSR. However, we did not find evidence to support a stronger effect for either of the two treatments. Offering therapy to perpetrators of intimate partner violence constitutes an important part of the protection of the victims. The amount of randomized controlled studies on the effectiveness of cognitive behaviour group therapy is still sparse and our study is an important contribution in this respect. With regard to studies of MBSR for perpetrators of intimate partner violence, the evidence is still to be considered as preliminary. Even so, the finding that MBSR was associated with a substantial reduction in violent behaviour is noteworthy whilst difficult to interpret. It should at least inspire exploration of any core elements that CBGT and MBSR have in common, to learn more about factors promoting behavioural change.

### Clinical trial registration

The trial was registered in ClinicalTrials.gov (registration no NCT01653860, July 2012).

## Supplementary information

**Additional file 1: Figure S1.** Estimated number of reported incidents of psychological violence at baseline (0), 6 months and 12 months’ follow-up. 95% Confidence intervals (vertical lines). Estimates based on a linear mixed model. **Table S1.**a Difference in mean number of reported incidents of psychological violence last 3 months according to time and intervention b Difference in mean number of reported incidents of physical violence last 3 months according to time and intervention c Difference in mean number of reported incidents of sexual violence last 3 months according to time and intervention d Difference in mean number of reported incidents of injury violence last 3 months according to time and intervention **Table S2.** a Odds ratio for physical violence last 3 months according to time and intervention b Odds ratio for psychological violence last 3 months according to time and intervention c Odds ratio for sexual violence last 3 months according to time and intervention d Odds ratio for injury last 3 months according to time and intervention Client scores: **Table S3.** Difference in mean number of reported incidents of psychological violence last 3 months according to time and intervention **Table S4.**a Odds ratio for physical violence last 3 months according to time and intervention b Odds ratio for psychological violence last 3 months according to time and intervention S-Table 4c Odds ratio for sexual violence last 3 months according to time and intervention d Odds ratio for injury last 3 months according to time and intervention **Table S5.**a Reported psychological violence the last 3 months at baseline, CBGT and MBSR, clients and partners combined **b** Reported psychological violence at time 2, CBGT and MBSR, clients and partners combined **c** Reported psychological violence at time 3, CBGT and MBSR, clients and partners combined **d** Reported psychological violence at time 4, CBGT and MBSR, clients and partners combined **e** Reported psychological violence at time 5, CBGT and MBSR, clients and partners combined **Table S6.a** Reported physical violence at baseline, CBGT and MBSR, clients and partners combined **b** Reported physical violence at time 2, CBGT and MBSR, clients and partners combined **c** Reported physical violence at time 3, CBGT and MBSR, clients and partners combined **d** Reported physical violence at time 4, CBGT and MBSR, clients and partners combined **e** Reported physical violence at time 5, CBGT and MBSR, clients and partners combined **Table S7.a** Reported sexual violence at baseline, CBGT and MBSR, clients and partners combined **b** Reported sexual violence at time 2, CBGT and MBSR, clients and partners combined **c** Reported sexual violence at time 3, CBGT and MBSR, clients and partners combined **d** Reported sexual violence at time 4, CBGT and MBSR, clients and partners combined **e** Reported sexual violence at time 5, CBGT and MBSR, clients and partners combined **Table S8.a** Reported injury on partner at baseline, CBGT and MBSR, clients and partners combined **b** Reported injury on partner at time 2, CBGT and MBSR, clients and partners combined **c** Reported injury on partner at time 3, CBGT and MBSR, clients and partners combined **d** Reported injury on partner at time 4, CBGT and MBSR, clients and partners combined **e** Reported injury on partner at time 5, CBGT and MBSR, clients and partners combined

## Data Availability

Data and materials are available on request.

## Abbreviations

CBT: Cognitive behaviour therapy
CBGT: Cognitive behavioural group therapy
CI: Confidence interval
CTS2: Revised conflict tactics scale
GEE: Generalized estimating equation
IPV: Intimate partner violence
MBSR: Mindfulness-based stress reduction
SD: Standard deviation
TAU: Treatment as usual
