# Supplementary material for: Cognitive behavioural group therapy versus mindfulness-based stress reduction group therapy for intimate partner violence: a randomized controlled trial
Source: BMC Psychiatry. 2020 Apr 19;20:178. doi: 10.1186/s12888-020-02582-4 (PMC7169006; doi:10.1186/s12888-020-02582-4)
Supplement: Supplementary file 1 — Additional file 1: Figure S1. Estimated number of reported incidents of psychological violence at baseline (0), 6 months and 12 months’ follow-up. 95% Confidence intervals (vertical lines). Estimates based on a linear mixed model. Table S1.a Difference in mean number of reported incidents of psychological violence last 3 months according to time and intervention b Difference in mean number of reported incidents of physical violence last 3 months according to time and intervention c Difference in mean number of reported incidents of sexual violence last 3 months according to time and intervention d Difference in mean number of reported incidents of injury violence last 3 months according to time and intervention Table S2. a Odds ratio for physical violence last 3 months according to time and intervention b Odds ratio for psychological violence last 3 months according to time and intervention c Odds ratio for sexual violence last 3 months according to time and intervention d Odds ratio for injury last 3 months according to time and intervention Client scores: Table S3. Difference in mean number of reported incidents of psychological violence last 3 months according to time and intervention Table S4.a Odds ratio for physical violence last 3 months according to time and intervention b Odds ratio for psychological violence last 3 months according to time and intervention S-Table 4c Odds ratio for sexual violence last 3 months according to time and intervention d Odds ratio for injury last 3 months according to time and intervention Table S5.a Reported psychological violence the last 3 months at baseline, CBGT and MBSR, clients and partners combined b Reported psychological violence at time 2, CBGT and MBSR, clients and partners combined c Reported psychological violence at time 3, CBGT and MBSR, clients and partners combined d Reported psychological violence at time 4, CBGT and MBSR, clients and partners combined e Reported psychological violence at time 5, CBGT and MBSR [file 12888_2020_2582_MOESM1_ESM.docx]

**Supplementary Fig. 1** Estimated number of reported incidents of psychological violence at baseline (0), 6 months and 12 months’ follow-up. 95% Confidence intervals (vertical lines). Estimates based on a linear mixed model.

**S-Table 1a** Difference in mean number of reported incidents of psychological violence last 3 months according to time and intervention

|  | **β^a^** | **95% CI** | **p-value** |
| --- | --- | --- | --- |
| CBGT compared with MBSR at start of treatment | 0.44 | -0.44 to 1.31 | 0.33 |
| Time 3-6 months compared with start of treatment - MBSR | -0.93 | -1.56 to -0.30 | 0.004 |
| Time 9-12 months compared with start of treatment - MBSR | -0.67 | -1.34 to -0.0001 | 0.05 |
| Time 3-6 months compared with start of treatment - CBGT | -0.62 | -1.28 to 0.04 | 0.07 |
| Time 9-12 months compared with start of treatment - CBGT | -1.01 | -1.65 to -0.36 | 0.002 |
| Time 3-6 months – additional intervention effect ^b^ | 0.31 | -0.61 to 1.23 | 0.51 |
| Time 9-12 months – additional intervention effect ^b^ | -0.34 | -1.27 to 0.60 | 0.48 |

Estimated difference in mean for psychological violence with 95% confidence intervals, using a linear mixed model. Based on a combination of highest reported level of violence from client or partner.

Estimated days per month with 95% confidence intervals. 380 observations in 125 clients.

^a^ Unstandardized regression coefficient.

^b^ Estimate for additional effect of time for the CBGT group compared with the MBSR group relative to start of treatment.

**S-Table 1b** Difference in mean number of reported incidents of physical violence last 3 months according to time and intervention

|  | **β^a^** | **95% CI** | **p-value** |
| --- | --- | --- | --- |
| CBGT compared with MBSR at start of treatment | -0.01 | -0.29 to 0.27 | 0.94 |
| Time 3-6 months compared with start of treatment - MBSR | -0.51 | -0.72 to -0.29 | <0.001 |
| Time 9-12 months compared with start of treatment - MBSR | -0.44 | -0.67 to -0.21 | <0.001 |
| Time 3-6 months compared with start of treatment - CBGT | -0.47 | -0.69 to -0.24 | <0.001 |
| Time 9-12 months compared with start of treatment - CBGT | -0.57 | -0.79 to -0.35 | <0.001 |
| Time 3-6 months – additional intervention effect ^b^ | 0.04 | -0.27 to 0.35 | 0.80 |
| Time 9-12 months – additional intervention effect ^b^ | -0.13 | -0.45 to 0.19 | 0.42 |

Estimated difference in mean for psychological violence with 95% confidence intervals, using a linear mixed model. Based on a combination of highest reported level of violence from client or partner.

Estimated days per month with 95% confidence intervals. 380 observations in 125 clients.

^a^ Unstandardized regression coefficient.

^b^ Estimate for additional effect of time for the CBGT group compared with the MBSR group relative to start of treatment.

**S-Table 1c** Difference in mean number of reported incidents of sexual violence last 3 months according to time and intervention

|  | **β^a^** | **95% CI** | **p-value** |
| --- | --- | --- | --- |
| CBGT compared with MBSR at start of treatment | 0.08 | -0.90 to 1.06 | 0.87 |
| Time 3-6 months compared with start of treatment - MBSR | -0.68 | -0.88 to -0.47 | <0.001 |
| Time 9-12 months compared with start of treatment - MBSR | -0.67 | -0.88 to -0.45 | <0.001 |
| Time 3-6 months compared with start of treatment - CBGT | -0.59 | -0.81 to -0.36 | <0.001 |
| Time 9-12 months compared with start of treatment - CBGT | -0.55 | -0.77 to -0.34 | <0.001 |
| Time 3-6 months – additional intervention effect ^b^ | 0.09 | -0.21 to 0.39 | 0.56 |
| Time 9-12 months – additional intervention effect ^b^ | 0.11 | -0.19 to 0.42 | 0.47 |

Estimated difference in mean for psychological violence with 95% confidence intervals, using a linear mixed model. Based on a combination of highest reported level of violence from client or partner.

Estimated days per month with 95% confidence intervals. 379 observations in 125 clients.

^a^ Unstandardized regression coefficient.

^b^ Estimate for additional effect of time for the CBGT group compared with the MBSR group relative to start of treatment.

**S-Table 1d** Difference in mean number of reported incidents of injury last 3 months according to time and intervention

|  | **β^a^** | **95% CI** | **p-value** |
| --- | --- | --- | --- |
| CBGT compared with MBSR at start of treatment | 0.05 | -0.28 to 0.38 | 0.76 |
| Time 3-6 months compared with start of treatment - MBSR | -0.66 | -0.99 to -0.34 | <0.001 |
| Time 9-12 months compared with start of treatment - MBSR | -0.67 | -1.01 to -0.33 | <0.001 |
| Time 3-6 months compared with start of treatment - CBGT | -0.46 | -0.78 to -0.13 | 0.006 |
| Time 9-12 months compared with start of treatment - CBGT | -0.73 | -1.05 to -0.42 | <0.001 |
| Time 3-6 months – additional intervention effect ^b^ | 0.21 | -0.25 to 0.66 | 0.38 |
| Time 9-12 months – additional intervention effect ^b^ | -0.06 | -0.52 to 0.40 | 0.79 |

Estimated difference in mean for psychological violence with 95% confidence intervals, using a linear mixed model. Based on a combination of highest reported level of violence from client or partner.

Estimated days per month with 95% confidence intervals. 380 observations in 125 clients.

^a^ Unstandardized regression coefficient.

^b^ Estimate for additional effect of time for the CBGT group compared with the MBSR group relative to start of treatment.

**S-Table 2a** Odds ratio for physical violence last 3 months according to time and intervention

|  | **OR** | **95% CI** | **p-value** |
| --- | --- | --- | --- |
| CBGT compared with MBSR at start of treatment | 0.80 | 0.28 to 2.26 | 0.68 |
| Time 3-6 months compared with start of treatment - MBSR | 0.03 | 0.01 to 0.08 | <.001 |
| Time 9-12 months compared with start of treatment - MBSR | 0.03 | 0.01 to 0.09 | <.001 |
| Time 3-6 months compared with start of treatment - CBGT | 0.02 | 0.007 to 0.05 | <.001 |
| Time 9-12 months compared with start of treatment - CBGT | 0.01 | 0.005 to 0.04 | <.001 |
| Time 3-6 months – additional intervention effect ^a^ | 0.56 | 0.14 to 2.14 | 0.39 |
| Time 9-12 months – additional intervention effect ^a^ | 0.42 | 0.10 to 1.74 | 0.23 |

Estimated OR for physical violence with 95% confidence intervals, using a generalized estimating equation (GEE) logistic regression model. Based on a combination of highest reported level of violence from client or partner. 380 observations in 125 clients.

^a^ Estimate for additional effect of time for the CBGT group compared with the MBSR group relative to start of treatment.

**S-Table 2b** Odds ratio for psychological violence last 3 months according to time and intervention

|  | **OR** | **95% CI** | **p-value** |
| --- | --- | --- | --- |
| CBGT compared with MBSR at start of treatment | 0.64 | 0.20 to 2.01 | 0.45 |
| Time 3-6 months compared with start of treatment - MBSR | 0.25 | 0.10 to 0.60 | 0.002 |
| Time 9-12 months compared with start of treatment - MBSR | 0.18 | 0.07 to 0.45 | <.001 |
| Time 3-6 months compared with start of treatment - CBGT | 0.57 | 0.26 to 1.26 | 0.16 |
| Time 9-12 months compared with start of treatment - CBGT | 0.33 | 0.16 to 0.70 | 0.004 |
| Time 3-6 months – additional intervention effect ^a^ | 2.27 | 0.69 to 7.42 | 0.18 |
| Time 9-12 months – additional intervention effect ^a^ | 1.83 | 0.57 to 5.86 | 0.31 |

Estimated OR for psychological violence with 95% confidence intervals, using a generalized estimating equation (GEE) logistic regression model. Based on a combination of highest reported level of violence from client or partner. 380 observations in 125 clients.

^a^ Estimate for additional effect of time for the CBGT group compared with the MBSR group relative to start of treatment.

**S-Table 2c** Odds ratio for sexual violence last 3 months according to time and intervention

|  | **OR** | **95% CI** | **p-value** |
| --- | --- | --- | --- |
| CBGT compared with MBSR at start of treatment | 0.60 | 0.29 to 1.22 | 0.16 |
| Time 3-6 months compared with start of treatment - MBSR | 0.04 | 0.01 to 0.12 | <.001 |
| Time 9-12 months compared with start of treatment - MBSR | 0.05 | 0.02 to 0.15 | <.001 |
| Time 3-6 months compared with start of treatment - CBGT | 0.08 | 0.03 to 0.24 | <.001 |
| Time 9-12 months compared with start of treatment - CBGT | 0.07 | 0.02 to 0.22 | <.001 |
| Time 3-6 months – additional intervention effect ^a^ | 2.04 | 0.42 to 10.02 | 0.38 |
| Time 9-12 months – additional intervention effect ^a^ | 1.48 | 0.31 to 7.12 | 0.63 |

Estimated OR for sexual violence with 95% confidence intervals, using a generalized estimating equation (GEE) logistic regression model. Based on a combination of highest reported level of violence from client or partner. 379 observations in 125 clients.

^a^ Estimate for additional effect of time for the CBGT group compared with the MBSR group relative to start of treatment.

**S-Table 2d** Odds ratio for injury last 3 months according to time and intervention

|  | **OR** | **95% CI** | **p-value** |
| --- | --- | --- | --- |
| CBGT compared with MBSR at start of treatment | 0.65 | 0.29 to 1.45 | 0.29 |
| Time 3-6 months compared with start of treatment - MBSR | 0.01 | 0.002 to 0.04 | <.001 |
| Time 9-12 months compared with start of treatment - MBSR | 0.02 | 0.005 to 0.06 | <.001 |
| Time 3-6 months compared with start of treatment - CBGT | 0.03 | 0.01 to 0.10 | <.001 |
| Time 9-12 months compared with start of treatment - CBGT | 0.02 | 0.003 to 0.06 | <.001 |
| Time 3-6 months – additional intervention effect ^a^ | 3.92 | 0.59 to 26.07 | 0.16 |
| Time 9-12 months – additional intervention effect ^a^ | 0.86 | 0.13 to 5.93 | 0.88 |

Estimated OR for injury with 95% confidence intervals, using a generalized estimating equation (GEE) logistic regression model. Based on a combination of highest reported level of violence from client or partner. 380 observations in 125 clients.

^a^ Estimate for additional effect of time for the CBGT group compared with the MBSR group relative to start of treatment.

**Client scores**

**S-Table 3** Difference in mean number of reported incidents of psychological violence last 3 months according to time and intervention

|  | **β^a^** | **95% CI** | **p-value** |
| --- | --- | --- | --- |
| CBGT compared with MBSR at start of treatment | 0.38 | -0.26 to 1.02 | 0.24 |
| Time 3-6 months compared with start of treatment - MBSR | -0.79 | -1.17 to -0.41 | <.001 |
| Time 9-12 months compared with start of treatment - MBSR | -0.79 | -1.19 to -0.39 | <.001 |
| Time 3-6 months compared with start of treatment - CBGT | -0.83 | -1.24 to -0.42 | <.001 |
| Time 9-12 months compared with start of treatment - CBGT | -1.02 | -1.42 to -0.62 | <.001 |
| Time 3-6 months – additional intervention effect ^b^ | -0.04 | -0.60 to 0.52 | 0.89 |
| Time 9-12 months – additional intervention effect ^b^ | -0.23 | -0.80 to 0.34 | 0.42 |

Estimated difference in mean for psychological violence with 95% confidence intervals, using a linear mixed model. Based on reported level of violence from client only. 355 observations in 125 patients.

Estimated days per month with 95% confidence intervals.

^a^ Unstandardized regression coefficient.

^b^ Estimate for additional effect of time for the CBGT group compared with the MBSR group relative to start of treatment.

**S-Table 4a** Odds ratio for physical violence last 3 months according to time and intervention

|  | **OR** | **95% CI** | **p-value** |
| --- | --- | --- | --- |
| CBGT compared with MBSR at start of treatment | 0.84 | 0.31 to 2.26 | 0.73 |
| Time 3-6 months compared with start of treatment MBSR | 0.02 | 0.01 to 0.06 | <.001 |
| Time 9-12 months compared with start of treatment - MBSR | 0.03 | 0.01 to 0.08 | <.001 |
| Time 3-6 months compared with start of treatment - CBGT | 0.02 | 0.01 to 0.06 | <.001 |
| Time 9-12 months compared with start of treatment - CBGT | 0.01 | 0.003-0.04 | <.001 |
| Time 3-6 months – additional intervention effect ^a^ | 0.95 | 0.21 to 4.37 | 0.95 |
| Time 9-12 months – additional intervention effect ^a^ | 0.39 | 0.07 to 2.06 | 0.27 |

Estimated OR for physical violence with 95% confidence intervals, using a generalized estimating equation (GEE) logistic regression model. Based on a combination of level of violence from client only. 355 observations in 125 clients.

^a^ Estimate for additional effect of time for the CBGT group compared with the MBSR group relative to start of treatment.

**S-Table 4b** Odds ratio for psychological violence last 3 months according to time and intervention

|  | **OR** | **95% CI** | **p-value** |
| --- | --- | --- | --- |
| CBGT compared with MBSR at start of treatment | 0.62 | 0.21 to 1.79 | 0.38 |
| Time 3-6 months compared with start of treatment - MBSR | 0.23 | 0.10 to 0.50 | <.001 |
| Time 9-12 months compared with start of treatment - MBSR | 0.19 | 0.08 to 0.42 | <.001 |
| Time 3-6 months compared with start of treatment - CBGT | 0.46 | 0.23 to 0.94 | 0.034 |
| Time 9-12 months compared with start of treatment - CBGT | 0.27 | 0.14 to 0.53 | <.001 |
| Time 3-6 months – additional intervention effect ^a^ | 2.04 | 0.71 to 5.89 | 0.19 |
| Time 9-12 months – additional intervention effect ^a^ | 1.43 | 0.50 to 4.10 | 0.51 |

Estimated OR for psychological violence with 95% confidence intervals, using a generalized estimating equation (GEE) logistic regression model. Based on the reported level of violence from client only. 355 observations in 125 clients.

^a^ Estimate for additional effect of time for the CBGT group compared with the MBSR group relative to start of treatment.

**S-Table 4c** Odds ratio for sexual violence last 3 months according to time and intervention

|  | **OR** | **95% CI** | **p-value** |
| --- | --- | --- | --- |
| CBGT compared with MBSR at start of treatment | 0.62 | 0.30 to 1.26 | 0.19 |
| Time 3-6 months compared with start of treatment - MBSR | 0.02 | 0.01 to 0.10 | <.001 |
| Time 9-12 months compared with start of treatment - MBSR | 0.01 | 0.002 to 0.10 | <.001 |
| Time 3-6 months compared with start of treatment - CBGT | 0.04 | 0.01 to 0.20 | <.001 |
| Time 9-12 months compared with start of treatment - CBGT | 0.06 | 0.02 to 0.22 | <.001 |
| Time 3-6 months – additional intervention effect ^a^ | 1.97 | 0.24 to 16.35 | 0.53 |
| Time 9-12 months – additional intervention effect ^a^ | 4.87 | 0.45 to 53.35 | 0.19 |

Estimated OR for sexual violence with 95% confidence intervals, using a generalized estimating equation (GEE) logistic regression model. Based on the reported level of violence from client only. 355 observations in 125 clients.

^a^ Estimate for additional effect of time for the CBGT group compared with the MBSR group relative to start of treatment.

**S-Table 4d** Odds ratio for injury last 3 months according to time and intervention

|  | **OR** | **95% CI** | **p-value** |
| --- | --- | --- | --- |
| CBGT compared with MBSR at start of treatment | 0.60 | 0.26 to 1.36 | 0.22 |
| Time 3-6 months compared with start of treatment - MBSR | 0.004 | 0.001 to 0.03 | <.001 |
| Time 9-12 months compared with start of treatment - MBSR | 0.01 | 0.001 to 0.04 | <.001 |
| Time 3-6 months compared with start of treatment - CBGT | 0.02 | 0.004 to 0.08 | <.001 |
| Time 9-12 months compared with start of treatment - CBGT | 0.01 | 0.001 to 0.06 | <.001 |
| Time 3-6 months – additional intervention effect ^a^ | 4.21 | 0.32 to 54.77 | 0.27 |
| Time 9-12 months – additional intervention effect ^a^ | 1.62 | 0.09 to 29.51 | 0.74 |
| Estimated OR for injury with 95% confidence intervals using a generalized estimating equation (GEE) logistic regression model. Based on the reported level of violence from client only. 355 observations in 125 clients.  ^a^ Estimate for additional effect of time for the CBGT group compared with the MBSR group relative to start of treatment. | | | |

**S-Table 5a** Reported psychological violence the last three months at baseline, CBGT and MBSR, clients and partners combined

| Mean score on the CTS2 | MBSR  N = 57 | CBGT  N = 67 | Total  N = 124 |
| --- | --- | --- | --- |
| 0 | 5 | 9 | 14 |
| 0.1 | 4 | 3 | 7 |
| 0.2 | 3 | 3 | 6 |
| 0.3 | 4 | 3 | 7 |
| 0.4 | 2 | 5 | 7 |
| 0.5 | 1 | 1 | 2 |
| 0.6 | 1 | 4 | 5 |
| 0.8 | 5 | 0 | 5 |
| 0.9 | 1 | 3 | 4 |
| 1 | 0 | 2 | 2 |
| 1.1 | 3 | 2 | 5 |
| 1.2 | 4 | 0 | 4 |
| 1.3 | 2 | 0 | 2 |
| 1.4 | 3 | 1 | 4 |
| 1.5 | 0 | 1 | 1 |
| 1.6 | 1 | 0 | 1 |
| 1.7 | 0 | 2 | 2 |
| 1.8 | 0 | 2 | 2 |
| 1.9 | 1 | 0 | 1 |
| 2 | 0 | 3 | 3 |
| 2.1 | 1 | 0 | 1 |
| 2.2 | 0 | 1 | 1 |
| 2.3 | 0 | 4 | 4 |
| 2.4 | 5 | 2 | 7 |
| 2.5 | 1 | 0 | 1 |
| 3.3 | 1 | 1 | 2 |
| 3.4 | 1 | 1 | 2 |
| 3.6 | 0 | 1 | 1 |
| 3.7 | 0 | 1 | 1 |
| 3.8 | 1 | 0 | 1 |
| 4 | 0 | 1 | 1 |
| 4.1 | 0 | 2 | 2 |
| 4.6 | 1 | 0 | 1 |
| 4.7 | 0 | 2 | 2 |
| 4.9 | 1 | 1 | 2 |
| 5.3 | 1 | 0 | 1 |
| 5.4 | 0 | 1 | 1 |
| 5.8 | 1 | 0 | 1 |
| 7.4 | 1 | 0 | 1 |
| 7.5 | 1 | 0 | 1 |
| 9.4 | 0 | 1 | 1 |
| 9.5 | 0 | 1 | 1 |
| 11.1 | 1 | 0 | 1 |
| 11.2 | 0 | 1 | 1 |
| 12.7 | 0 | 1 | 1 |
| 13.1 | 0 | 1 | 1 |

*CBGT* Cognitive Behaviour Group Therapy; *MBSR* Mindfulness-Based Stress Reduction. *CTS2* Conflict Tactics Scales Revised

**S-Table 5b** Reported psychological violence at time 2, CBGT and MBSR, clients and partners combined

| Mean score on the CTS2 | MBSR  N = 46 | CBGT  N = 30 | Total  N = 76 |
| --- | --- | --- | --- |
| 0 | 13 | 6 | 19 |
| 0.1 | 3 | 3 | 6 |
| 0.2 | 1 | 1 | 2 |
| 0.3 | 2 | 1 | 3 |
| 0.4 | 2 | 2 | 4 |
| 0.5 | 8 | 2 | 10 |
| 0.6 | 3 | 1 | 4 |
| 0.7 | 2 | 1 | 3 |
| 0.8 | 1 | 2 | 3 |
| 1 | 2 | 3 | 5 |
| 1.1 | 2 | 0 | 2 |
| 1.2 | 0 | 1 | 1 |
| 1.3 | 0 | 1 | 1 |
| 1.6 | 0 | 1 | 1 |
| 2.1 | 0 | 1 | 1 |
| 2.6 | 2 | 0 | 2 |
| 4 | 1 | 0 | 1 |
| 4.2 | 0 | 1 | 1 |
| 5 | 1 | 0 | 1 |
| 5.2 | 1 | 0 | 1 |
| 6 | 0 | 1 | 1 |
| 9 | 1 | 0 | 1 |
| 9.6 | 0 | 1 | 1 |
| 10 | 1 | 0 | 1 |
| 13.6 | 0 | 1 | 1 |

*CBGT* Cognitive Behaviour Group Therapy; *MBSR* Mindfulness-Based Stress Reduction. *CTS2* Conflict Tactics Scales Revised

**S-Table 5c** Reported psychological violence at time 3, CBGT and MBSR, clients and partners combined

| Mean score on the CTS2 | MBSR  N = 25 | CBGT  N = 29 | Total  N = 54 |
| --- | --- | --- | --- |
| 0 | 8 | 7 | 15 |
| 0.1 | 4 | 4 | 8 |
| 0.2 | 3 | 0 | 3 |
| 0.3 | 1 | 2 | 3 |
| 0.4 | 2 | 0 | 2 |
| 0.5 | 1 | 4 | 5 |
| 0.6 | 0 | 1 | 1 |
| 0.7 | 0 | 1 | 1 |
| 0.8 | 0 | 1 | 1 |
| 0.9 | 0 | 1 | 1 |
| 1.2 | 1 | 1 | 2 |
| 1.3 | 2 | 0 | 2 |
| 1.7 | 1 | 0 | 1 |
| 2.1 | 0 | 1 | 1 |
| 2.6 | 0 | 2 | 2 |
| 3.2 | 0 | 1 | 1 |
| 3.3 | 1 | 0 | 1 |
| 4.3 | 0 | 1 | 1 |
| 7.3 | 1 | 0 | 1 |
| 10.3 | 0 | 1 | 1 |
| 15 | 0 | 1 | 1 |

*CBGT* Cognitive Behaviour Group Therapy; *MBSR* Mindfulness-Based Stress Reduction. *CTS2* Conflict Tactics Scales Revised

**S-Table 5d** Reported psychological violence at time 4, CBGT and MBSR, clients and partners combined

| Mean score on the CTS2 | MBSR  N = 26 | CBGT  N = 31 | Total  N = 57 |
| --- | --- | --- | --- |
| 0 | 8 | 7 | 15 |
| 0.1 | 1 | 3 | 4 |
| 0.2 | 0 | 4 | 4 |
| 0.3 | 0 | 2 | 2 |
| 0.4 | 4 | 3 | 7 |
| 0.5 | 1 | 0 | 1 |
| 0.6 | 2 | 0 | 2 |
| 0.8 | 2 | 0 | 2 |
| 0.9 | 2 | 0 | 2 |
| 1.1 | 0 | 2 | 2 |
| 1.2 | 1 | 3 | 4 |
| 1.4 | 1 | 0 | 1 |
| 1.5 | 1 | 1 | 2 |
| 2.3 | 0 | 1 | 1 |
| 2.8 | 0 | 1 | 1 |
| 3 | 0 | 1 | 1 |
| 3.7 | 0 | 1 | 1 |
| 6.5 | 1 | 0 | 1 |
| 7.8 | 0 | 1 | 1 |
| 8.1 | 1 | 0 | 1 |
| 9.6 | 1 | 0 | 1 |
| 9.9 | 0 | 1 | 1 |

*CBGT* Cognitive Behaviour Group Therapy; *MBSR* Mindfulness-Based Stress Reduction. *CTS2* Conflict Tactics Scales Revised

**S-Table 5e** Reported psychological violence at time 5, CBGT and MBSR, clients and partners combined

| Mean score on the CTS2 | MBSR  N = 33 | CBGT  N = 36 | Total  N = 69 |
| --- | --- | --- | --- |
| 0 | 13 | 15 | 28 |
| 0.1 | 0 | 6 | 6 |
| 0.2 | 4 | 0 | 4 |
| 0.3 | 3 | 3 | 6 |
| 0.4 | 1 | 1 | 2 |
| 0.5 | 2 | 0 | 2 |
| 0.7 | 3 | 1 | 4 |
| 0.8 | 1 | 2 | 3 |
| 0.9 | 0 | 2 | 2 |
| 1 | 1 | 1 | 2 |
| 1.4 | 1 | 0 | 1 |
| 1.7 | 0 | 1 | 1 |
| 2.5 | 1 | 0 | 1 |
| 2.7 | 0 | 1 | 1 |
| 4.8 | 1 | 0 | 1 |
| 6.5 | 0 | 1 | 1 |
| 6.6 | 1 | 0 | 1 |
| 7.2 | 0 | 1 | 1 |
| 9.3 | 0 | 1 | 1 |
| 9.7 | 1 | 0 | 1 |
|  |  |  |  |

*CBGT* Cognitive Behaviour Group Therapy; *MBSR* Mindfulness-Based Stress Reduction. *CTS2* Conflict Tactics Scales Revised

**S-Table 6a** Reported physical violence at baseline, CBGT and MBSR, clients and partners combined

| Mean score on the CTS2 | MBSR  N = 57 | CBGT  N = 67 | Total  N = 124 |
| --- | --- | --- | --- |
| 0 | 7 | 10 | 17 |
| 0.08 | 4 | 2 | 6 |
| 0.16 | 3 | 9 | 12 |
| 0.3 | 16 | 16 | 32 |
| 0.4 | 3 | 0 | 3 |
| 0.5 | 2 | 1 | 3 |
| 0.58 | 0 | 2 | 2 |
| 0.66 | 1 | 9 | 10 |
| 0.75 | 3 | 1 | 4 |
| 0.83 | 1 | 2 | 3 |
| 0.91 | 0 | 1 | 1 |
| 1 | 2 | 0 | 2 |
| 1.25 | 9 | 3 | 12 |
| 1.33 | 0 | 1 | 1 |
| 1.4 | 1 | 0 | 1 |
| 1.5 | 0 | 2 | 2 |
| 1.58 | 0 | 1 | 1 |
| 1.6 | 1 | 0 | 1 |
| 2.08 | 4 | 5 | 9 |
| 2.16 | 0 | 1 | 1 |
| 2.3 | 0 | 1 | 1 |

*CBGT* Cognitive Behaviour Group Therapy; *MBSR* Mindfulness-Based Stress Reduction. *CTS2* Conflict Tactics Scales Revised

**S-Table 6b** Reported physical violence at time 2, CBGT and MBSR, clients and partners combined

| Mean score on the CTS2 | MBSR  N = 46 | CBGT  N = 30 | Total  N = 76 |
| --- | --- | --- | --- |
| 0 | 37 | 25 | 62 |
| 0.08 | 3 | 1 | 4 |
| 0.09 | 1 | 0 | 1 |
| 0.16 | 1 | 1 | 2 |
| 0.25 | 2 | 0 | 2 |
| 0.33 | 0 | 1 | 1 |
| 0.41 | 1 | 0 | 1 |
| 0.5 | 0 | 1 | 1 |
| 0.9 | 1 | 0 | 1 |
| 2 | 0 | 1 | 1 |

*CBGT* Cognitive Behaviour Group Therapy; *MBSR* Mindfulness-Based Stress Reduction. *CTS2* Conflict Tactics Scales Revised

**S-Table 6c** Reported physical violence at time 3, CBGT and MBSR, clients and partners combined

| Mean score on the CTS2 | MBSR  N = 25 | CBGT  N = 29 | Total  N = 54 |
| --- | --- | --- | --- |
| 0 | 20 | 28 | 48 |
| 0.09 | 1 | 0 | 1 |
| 0.16 | 1 | 0 | 1 |
| 0.5 | 1 | 0 | 1 |
| 1.5 | 1 | 0 | 1 |
| 6.5 | 1 | 0 | 1 |
| 8.1 | 0 | 1 | 1 |

*CBGT* Cognitive Behaviour Group Therapy; *MBSR* Mindfulness-Based Stress Reduction. *CTS2* Conflict Tactics Scales Revised

**S-Table 6d** Reported physical violence at time 4, CBGT and MBSR, clients and partners combined

| Mean score on the CTS2 | MBSR  N = 26 | CBGT  N = 31 | Total  N = 57 |
| --- | --- | --- | --- |
| 0 | 21 | 29 | 50 |
| 0.08 | 2 | 0 | 2 |
| 0.16 | 0 | 1 | 1 |
| 0.25 | 1 | 0 | 1 |
| 0.33 | 1 | 0 | 1 |
| 0.5 | 0 | 1 | 1 |
| 8 | 1 | 0 | 1 |

*CBGT* Cognitive Behaviour Group Therapy; *MBSR* Mindfulness-Based Stress Reduction. *CTS2* Conflict Tactics Scales Revised

**S-Table 6e** Reported physical violence at time 5, CBGT and MBSR, clients and partners combined

| Mean score on the CTS2 | MBSR  N = 33 | CBGT  N = 36 | Total  N = 69 |
| --- | --- | --- | --- |
| 0 | 27 | 33 | 60 |
| 0.08 | 2 | 1 | 3 |
| 0.16 | 1 | 0 | 1 |
| 0.25 | 0 | 1 | 1 |
| 0.33 | 2 | 0 | 2 |
| 0.58 | 0 | 1 | 1 |
| 0.75 | 1 | 0 | 1 |

*CBGT* Cognitive Behaviour Group Therapy; *MBSR* Mindfulness-Based Stress Reduction. *CTS2* Conflict Tactics Scales Revised

**S-Table 7a** Reported sexual violence at baseline, CBGT and MBSR, clients and partners combined

| Mean score on the CTS2 | MBSR  N = 57 | CBGT  N = 66 | Total  N = 123 |
| --- | --- | --- | --- |
| 0 | 23 | 35 | 58 |
| 0.3 | 12 | 7 | 19 |
| 0.6 | 10 | 10 | 20 |
| 1 | 1 | 2 | 3 |
| 1.3 | 3 | 4 | 7 |
| 1.6 | 0 | 2 | 2 |
| 2 | 2 | 1 | 3 |
| 2.6 | 3 | 2 | 5 |
| 3 | 2 | 0 | 2 |
| 4 | 0 | 1 | 1 |
| 5 | 0 | 1 | 1 |
| 5.3 | 1 | 0 | 1 |
| 9 | 0 | 1 | 1 |

*CBGT* Cognitive Behaviour Group Therapy; *MBSR* Mindfulness-Based Stress Reduction. *CTS2* Conflict Tactics Scales Revised

**S-Table 7b** Reported sexual violence at time 2, CBGT and MBSR, clients and partners combined

| Mean score on the CTS2 | MBSR  N = 46 | CBGT  N = 30 | Total  N = 76 |
| --- | --- | --- | --- |
| 0 | 43 | 27 | 70 |
| 0.3 | 1 | 1 | 2 |
| 0.6 | 0 | 2 | 2 |
| 1.3 | 1 | 0 | 1 |
| 16.6 | 1 | 0 | 1 |

*CBGT* Cognitive Behaviour Group Therapy; *MBSR* Mindfulness-Based Stress Reduction. *CTS2* Conflict Tactics Scales Revised

**S-Table 7c** Reported sexual violence at time 3, CBGT and MBSR, clients and partners combined

| Mean score on the CTS2 | MBSR  N = 25 | CBGT  N = 29 | Total  N = 54 |
| --- | --- | --- | --- |
| 0 | 24 | 28 | 52 |
| 1 | 1 | 0 | 1 |
| 25 | 0 | 1 | 1 |

*CBGT* Cognitive Behaviour Group Therapy; *MBSR* Mindfulness-Based Stress Reduction. *CTS2* Conflict Tactics Scales Revised

**S-Table 7d** Reported sexual violence at time 4, CBGT and MBSR, clients and partners combined

| Mean score on the CTS2 | MBSR  N = 26 | CBGT  N = 31 | Total  N = 57 |
| --- | --- | --- | --- |
| 0 | 24 | 28 | 52 |
| 0.3 | 2 | 2 | 4 |
| 1.3 | 0 | 1 | 1 |

*CBGT* Cognitive Behaviour Group Therapy; *MBSR* Mindfulness-Based Stress Reduction. *CTS2* Conflict Tactics Scales Revised

**S-Table 7e** Reported sexual violence at time 5, CBGT and MBSR, clients and partners combined

| Mean score on the CTS2 | MBSR  N = 33 | CBGT  N = 36 | Total  N = 69 |
| --- | --- | --- | --- |
| 0 | 31 | 35 | 66 |
| 0.3 | 1 | 0 | 1 |
| 0.6 | 1 | 0 | 1 |
| 1.3 | 0 | 1 | 1 |

*CBGT* Cognitive Behaviour Group Therapy; *MBSR* Mindfulness-Based Stress Reduction. *CTS2* Conflict Tactics Scales Revised

**S-Table 8a** Reported injury on partner at baseline, CBGT and MBSR, clients and partners combined

| Mean score on the CTS2 | MBSR  N = 57 | CBGT  N = 67 | Total  N = 124 |
| --- | --- | --- | --- |
| 0 | 13 | 21 | 34 |
| 0.16 | 12 | 7 | 19 |
| 0.3 | 11 | 8 | 19 |
| 0.6 | 8 | 13 | 21 |
| 0.8 | 1 | 1 | 2 |
| 1.3 | 7 | 8 | 15 |
| 1.5 | 0 | 1 | 1 |
| 1.6 | 0 | 1 | 1 |
| 2.5 | 3 | 3 | 6 |
| 2.6 | 1 | 0 | 1 |
| 2.8 | 0 | 1 | 1 |
| 4.16 | 0 | 3 | 3 |
| 8.3 | 1 | 0 | 1 |

*CBGT* Cognitive Behaviour Group Therapy; *MBSR* Mindfulness-Based Stress Reduction. *CTS2* Conflict Tactics Scales Revised

**S-Table 8b** Reported injury on partner at time 2, CBGT and MBSR, clients and partners combined

| Mean score on the CTS2 | MBSR  N = 46 | CBGT  N = 30 | Total  N = 76 |
| --- | --- | --- | --- |
| 0 | 46 | 28 | 74 |
| 0.16 | 0 | 2 | 2 |

*CBGT* Cognitive Behaviour Group Therapy; *MBSR* Mindfulness-Based Stress Reduction. *CTS2* Conflict Tactics Scales Revised

**S-Table 8c** Reported injury on partner at time 3, CBGT and MBSR, clients and partners combined

| Mean score on the CTS2 | MBSR  N = 25 | CBGT  N = 29 | Total  N = 54 |
| --- | --- | --- | --- |
| 0 | 23 | 27 | 50 |
| 1 | 1 | 0 | 1 |
| 1.3 | 1 | 0 | 1 |
| 4.6 | 0 | 1 | 1 |
| 12.5 | 0 | 1 | 1 |

*CBGT* Cognitive Behaviour Group Therapy; *MBSR* Mindfulness-Based Stress Reduction. *CTS2* Conflict Tactics Scales Revised

**S-Table 8d** Reported injury on partner at time 4, CBGT and MBSR, clients and partners combined

| Mean score on the CTS2 | MBSR  N = 26 | CBGT  N = 31 | Total  N = 57 |
| --- | --- | --- | --- |
| 0 | 25 | 30 | 55 |
| 0.5 | 0 | 1 | 1 |
| 1 | 1 | 0 | 1 |

*CBGT* Cognitive Behaviour Group Therapy; *MBSR* Mindfulness-Based Stress Reduction. *CTS2* Conflict Tactics Scales Revised

**S-Table 8e** Reported injury on partner at time 5, CBGT and MBSR, clients and partners combined

| Mean score on the CTS2 | MBSR  N = 33 | CBGT  N = 36 | Total  N = 69 |
| --- | --- | --- | --- |
| 0 | 31 | 35 | 66 |
| 0.16 | 1 | 1 | 2 |
| 0.3 | 1 | 0 | 1 |

*CBGT* Cognitive Behaviour Group Therapy; *MBSR* Mindfulness-Based Stress Reduction. *CTS2* Conflict Tactics Scales Revised
